# Avelumab first-line maintenance treatment in patients with locally advanced or metastatic urothelial carcinoma: real-world results from a Korean expanded access program

**DOI:** 10.3389/fonc.2024.1403120

**Published:** 2024-06-03

**Authors:** Se Hoon Park, Sang Joon Shin, Sun Young Rha, Seung-Hoon Beom, Ho Kyung Seo, Bhumsuk Keam, Miso Kim, Yoon-Hee Hong, Shinkyo Yoon, Jae-Lyun Lee

**Affiliations:** ^1^ Department of Medicine, Samsung Medical Center, Sungkyunkwan University School of Medicine, Seoul, Republic of Korea; ^2^ Yonsei Cancer Center, Yonsei University College of Medicine, Seoul, Republic of Korea; ^3^ Department of Urology, Center for Urologic Cancer, National Cancer Center, Goyang, Republic of Korea; ^4^ Department of Internal Medicine, Seoul National University Hospital, Seoul, Republic of Korea; ^5^ Merck Ltd., Seoul, Republic of Korea; ^6^ Department of Oncology, Asan Medical Center, University of Ulsan College of Medicine, Seoul, Republic of Korea

**Keywords:** urothelial carcinoma, avelumab, maintenance treatment, platinum-based chemotherapy, real-world

## Abstract

**Background:**

The JAVELIN Bladder 100 phase 3 trial demonstrated the efficacy and safety of avelumab administered as first-line (1L) maintenance treatment in patients with advanced urothelial carcinoma (UC) without disease progression after 1L platinum-based chemotherapy. This study provides the first real-world data from Korea regarding avelumab 1L maintenance treatment, comprising data obtained from a nationwide expanded access program (EAP).

**Methods:**

This open-label EAP was conducted at five centers from September 2021 until June 2023. Eligible patients had unresectable locally advanced or metastatic UC and were progression free after 1L platinum-based chemotherapy. Patients received avelumab 10 mg/kg intravenously every 2 weeks per local prescribing information. Safety and effectiveness were assessed by treating physicians according to routine practice.

**Results:**

Overall, 30 patients were enrolled. At initial UC diagnosis, 20 patients (66.7%) had stage 4 disease and 12 (40.0%) had visceral metastases. The most common 1L chemotherapy regimen was gemcitabine + cisplatin (21 patients; 70.0%). All but one patient (96.7%) had received 4-6 cycles of 1L chemotherapy. The median interval from end of 1L chemotherapy to start of avelumab was 4.4 weeks. Median duration of avelumab treatment was 6.2 months (range, 0.9-20.7); nine patients (30.0%) received >12 months of treatment. Adverse events related to avelumab occurred in 21 patients (70.0%) and were grade ≥3 or classified as serious in three patients (10.0%). Median progression-free survival was 7.9 months (95% CI, 4.3-13.1). Overall survival was not analyzed because only one patient died.

**Conclusion:**

Results from this EAP demonstrated the clinical activity and acceptable safety of avelumab 1L maintenance treatment in Korean patients with advanced UC, consistent with previous studies.

## 1 Introduction

Urothelial carcinoma (UC) is a cancer that arises from the cells lining the urinary tract (1, 2). At least 90% of cases of UC originate in the bladder, with other cases originating in the ureter, urethra, or renal pelvis (1–3). Globally in 2020, bladder cancer was the tenth most common cancer and the thirteenth leading cause of death (4). The incidence of bladder cancer in Korea in 2020 (age-standardized rates per 100,000) according to the Korea National Cancer Incidence Database (KNCI DB) and International Agency for Research on Cancer (IARC), respectively, was 7.5 and 7.9 in men and 1.4 and 1.5 women. In comparison, global incidence rates (per IARC data) were 9.5 in men and 2.4 in women (5, 6). In Korea (per KNCI DB data), the annual incidence of bladder cancer increased nearly three-fold from 2000 to 2020 (from 1744 to 4753 cases). The incidence was five times higher in men than in women. During the same period, bladder cancer–related deaths per year in Korea doubled (from 778 to 1593) (7). The 5-year survival rate for patients diagnosed with bladder cancer in Korea from 2016 to 2020 was 76.5% (5).

Clinical stages of bladder cancer include nonmuscle-invasive, muscle-invasive, and locally advanced/metastatic (generally called advanced) disease, each of which exhibits distinct clinical characteristics and prognoses and requires different treatment approaches (2, 8). Although only ≈5% of patients are initially diagnosed with metastatic disease (2, 3), a notable proportion of patients diagnosed with earlier-stage disease will eventually progress to advanced disease. For example, ≈15% to 20% of nonmuscle-invasive tumors progress to muscle-invasive tumors, and even with radical cystectomy and pelvic lymph-node dissection, ≈50% of patients with muscle-invasive tumors will develop advanced disease (8). Metastatic bladder cancer has a poor prognosis, including a historical median overall survival (OS) of 13 to 15 months (2). In men in Korea diagnosed with bladder cancer with distant metastatic disease, the 5-year survival rate was 13.0% (data in women were not reported) (5).

For decades, platinum-based chemotherapy has been a standard first-line (1L) treatment for patients with advanced UC (2, 3, 8–10). In the early 1990s, the combination of methotrexate, vinblastine, doxorubicin, and cisplatin (MVAC) demonstrated increased response rates and longer OS compared with single-agent cisplatin, albeit with increased toxicity (11). Subsequently, cisplatin + gemcitabine became widely used following a randomized phase 3 trial showing similar effectiveness to MVAC but with improved tolerability (12, 13). Additionally, standard-dose MVAC was superseded by a dose-dense regimen that showed improved efficacy and tolerability (14, 15). Carboplatin + gemcitabine is the recommended 1L platinum regimen for patients unfit for cisplatin-based regimens (3, 9, 10, 16, 17).

In patients without progressive disease (PD) following platinum-based chemotherapy, avelumab (an immune checkpoint inhibitor) administered as 1L maintenance treatment is the recommended standard of care in international guidelines based on results from the pivotal JAVELIN Bladder 100 phase 3 trial (NCT02603432) (3, 9, 10, 18, 19). In this trial, 700 patients with advanced UC who were without PD following 4 to 6 cycles of 1L cisplatin + gemcitabine or carboplatin + gemcitabine were randomized to receive avelumab 1L maintenance + best supportive care (BSC) or BSC alone. After ≥2 years of follow-up, median OS was 23.8 vs 15.0 months (hazard ratio [HR], 0.76 [95% CI, 0.63-0.91]; p=0.0036) and median progression-free survival (PFS) was 5.5 vs 2.1 months (HR, 0.54 [95% CI, 0.46-0.64]; p<0.0001), respectively. The long-term safety of avelumab 1L maintenance treatment was also demonstrated and was consistent with previous analyses (19). In a *post hoc* analysis, median OS with avelumab 1L maintenance treatment measured from the start of 1L platinum-based chemotherapy in this population without PD was 29.7 months (20). In a subgroup analysis of patients enrolled in Asian countries, efficacy and safety findings were generally consistent with those observed in the overall population (21). Furthermore, prospective and retrospective real-world studies conducted in several countries have confirmed the favorable clinical activity and acceptable safety profile of avelumab 1L maintenance treatment reported in the JAVELIN Bladder 100 trial (22–25).

In Korea, avelumab was initially approved by the Ministry of Food and Drug Safety for the treatment of metastatic Merkel cell carcinoma on March 22, 2019, and it was subsequently approved for 1L maintenance treatment of patients with advanced UC on August 5, 2021. Here, we report the first real-world data from Korea in patients with advanced UC receiving avelumab 1L maintenance treatment, comprising data obtained from an expanded access program (EAP).

## 2 Materials and methods

### 2.1 Study design

The protocol for this nationwide, open-label EAP was approved on July 2, 2021, before the Korean approval for avelumab 1L maintenance treatment for patients with advanced UC on August 5, 2021. The EAP began on September 2, 2021, and continued until the final visit of the last patient on June 16, 2023. The purpose of the EAP was to offer access to avelumab 1L maintenance treatment before it was reimbursed in Korea. All patients were enrolled prospectively. Safety and effectiveness data were collected. The EAP was performed in accordance with ethical principles that have their origin in the Declaration of Helsinki and are consistent with International Council for Harmonisation and Good Clinical Practice and Ministry of Food and Drug Safety requirements. The protocol, amendments, and informed consent form were approved by institutional review boards at each site. Written informed consent was obtained from all patients.

### 2.2 Patients and treatment

Eligible patients were aged ≥18 years; had histologically confirmed, unresectable locally advanced or metastatic UC; and were progression free following 1L platinum-based chemotherapy. Patients were required to have adequate bone marrow function (absolute neutrophil count, ≥1500/mm^3^ or ≥1.5×10^9^/L; platelets, ≥100,000/mm^3^ or ≥100×10^9^/L; and hemoglobin, ≥9 g/dL); adequate renal function (estimated creatinine clearance, ≥30 mL/min); and adequate liver function (total serum bilirubin, ≤1.5× upper limit of normal [ULN]; aspartate aminotransferase and alanine aminotransferase, ≤2.5×ULN or, for patients with metastatic disease in the liver, ≤5×ULN). Patients were ineligible if they had hypersensitivity to avelumab or any of its excipients; had participated in clinical trials with avelumab; or had any severe acute or chronic medical condition inappropriate for entry into EAP, as determined by the treating physician.

Patients were treated with avelumab 10 mg/kg intravenously every 2 weeks per local prescribing information. Patients received premedication with an antihistamine and acetaminophen before the first 4 infusions of avelumab. After completing the fourth infusion without an infusion-related reaction, premedication for subsequent doses was administered at the discretion of the treating physician. Treatment continued until PD, unacceptable toxicity, or reimbursement approval or for 18 months from the last patient’s first visit, whichever occurred first.

### 2.3 Assessments

Baseline data, including demographics, medical and disease history, and prior treatment, were collected upon program enrollment (baseline). During avelumab treatment, clinical information, including data related to safety and effectiveness, was collected by treating physicians according to local routine practice. Data on avelumab exposure, safety, and effectiveness were obtained from the patient’s medical records from enrollment until last follow-up or data cutoff. Information on subsequent treatment was also collected.

Safety was monitored closely and recorded by the treating physician per local practice. Although data on all adverse events (AEs) were collected, only treatment-emergent AEs (TEAEs), i.e. those that initially occurred or worsened in severity or seriousness during the on-treatment period, were analyzed. The on-treatment period was defined as the time from the start date of avelumab treatment until the date of any of the following, whichever occurred first: final avelumab administration plus 30 days, termination of or discontinuation from the EAP, death, or administration of first subsequent anticancer therapy was received minus 1 day. Treatment-related adverse events (TRAEs) were defined as TEAEs that were classified by the treating physician as causally related to avelumab. TEAEs were coded using the Medical Dictionary for Regulatory Activities version 26.0, and severity was graded according to National Cancer Institute Common Terminology Criteria for Adverse Events version 4.03.

Effectiveness data collected included: survival status; PFS (defined as time from start of avelumab until the first documentation of PD or death from any cause, whichever occurred first); best overall response (confirmed complete response [CR], partial response [PR], stable disease [SD], PD, or not evaluable [NE]); objective response (confirmed CR or PR); disease control (confirmed CR, PR, or SD); time to response (defined as time from first avelumab administration until the first documentation of objective response); and duration of response (defined as time from the first documentation of objective response to the first documentation of PD or death from any cause, whichever occurred first). Tumor assessments were performed per local practice according to Response Evaluation Criteria in Solid Tumors version 1.1; the protocol did not include any specifications regarding the imaging modality to be used. Best response was determined using the following definitions: CR was defined as ≥2 determinations of CR recorded ≥4 weeks apart and before the first documentation of PD; PR was defined as ≥2 determinations of PR or better (PR followed by PR or PR followed by CR) recorded ≥4 weeks apart and before the first documentation of PD (and not qualifying as CR); SD was defined as ≥1 determination of SD (or better) ≥6 weeks after the start of first date of study drug administration and before the first documentation of PD (and not qualifying as CR or PR); PD was defined as the first documentation of PD ≤12 weeks after the first date of study drug administration (and not qualifying as CR, PR, or SD); all other cases were defined as NE.

### 2.4 Statistical analysis

For continuous variables, mean, standard deviation, median, minimum, and maximum were summarized in patients with nonmissing data. Categorical variables were summarized as number and percentage in patients with nonmissing data. PFS and duration of response were estimated using the Kaplan-Meier method, and corresponding 95% CIs for the median were calculated using the Brookmeyer-Crowley method with log-log transformation. Subgroup analyses of outcomes are not reported because of the small population size.

## 3 Results

### 3.1 Patients and treatment

Between September 28, 2021, and June 16, 2023, 31 patients provided informed consent and 30 patients were treated with avelumab at five centers in Korea (**Supplementary Table S1**); one patient did not receive avelumab treatment because they did not meet the eligibility criterion requiring lack of PD after 1L platinum-based chemotherapy. Baseline characteristics are summarized in **Table 1**.

**Table 1:** Baseline characteristics.

Characteristic	N=30
Age, years	
Mean (SD)	66.5 (9.5)
Median	65.5
Range	44.0-86.0
Sex, n (%)	
Male	26 (86.7)
Female	4 (13.3)
Weight, kg	
Mean (SD)	67.3 (12.0)
Range	47.0-93.6
ECOG PS, n (%)	
0	18 (60.0)
1	12 (40.0)
Years since diagnosis	
Mean (SD)	2.5 (2.8)
Median	1.3
Range	0.3-13.7
Primary tumor site, n (%)	
Upper tract only	7 (23.3)
Bladder only	21 (70.0)
Upper tract and bladder	1 (3.3)
Not reported	1 (3.3)
Disease stage at diagnosis, (%)	
1	2 (6.7)
2	1 (3.3)
3	2 (6.7)
4	20 (66.7)
Not reported	5 (16.7)
Tumor classification at diagnosis, n (%)	
*De novo* metastatic	19 (63.3)
Unresectable locally advanced	1 (3.3)
Eligible for curative surgery	10 (33.3)
Metastatic sites at diagnosis, n (%)	
Visceral only	7 (23.3)
Nonvisceral only	7 (23.3)
Visceral and nonvisceral	5 (16.7)
Received chemotherapy for earlier-stage disease, n (%)	
Yes	7 (23.3)
Neoadjuvant	5 (16.7)
Adjuvant	2 (6.7)
No	23 (76.7)
Received prior radiotherapy	
Yes	7 (23.3)
No	23 (76.7)
Underwent prior surgery	
Yes	24 (80.0)
No	6 (20.0)

ECOG PS, Eastern Cooperative Oncology Group performance status; SD, standard deviation.

The median time between initial UC diagnosis and enrollment was 1.3 years (range, 0.3-13.7). At initial UC diagnosis, the primary tumor location was bladder in 21 patients (70.0%), upper tract in 7 patients (23.3%), both bladder and upper tract in 1 patient (3.3%), and not reported in 1 patient (3.3%); disease stage was 1, 2, 3, or 4 in two patients (6.7%), one patient (3.3%), two patients (6.7%), and 20 patients (66.7%), respectively, and not reported in 5 patients (16.7%). Also at initial diagnosis, 10 patients (33.3%) were eligible for curative surgery, one patient (3.3%) had unresectable locally advanced disease, and 19 patients (63.3%) had distant metastases, including visceral and nonvisceral metastases in 12 (40.0%) and seven (23.3%), respectively. Seven patients (23.3%) had received chemotherapy for earlier stages of disease, including neoadjuvant chemotherapy in five patients (16.7%) and adjuvant chemotherapy in two patients (6.7%). In addition, seven patients (23.3%) had received radiation therapy, and 24 patients (80.0%) had undergone surgery, most commonly transurethral bladder resection (**Supplementary Table S2**).

At the start of avelumab 1L maintenance treatment, all patients had advanced UC, had received 1L platinum-based chemotherapy, and were progression free. Median age at the start of avelumab treatment was 65.5 years (range, 44-86), and Eastern Cooperative Oncology Group performance status was 0 in 18 patients (60.0%) and 1 in 12 patients (40.0%). Most patients were male (n=26 [86.7%]). The 1L chemotherapy regimen was cisplatin + gemcitabine in 21 patients (70.0%), carboplatin + gemcitabine in eight patients (26.7%), and MVAC in one patient (3.3%; **Table 2**). The number of cycles of 1L chemotherapy received was 4 in nine patients (30.0%), 5 in seven patients (23.3%), 6 in 13 patients (43.3%), and 8 in one patient (3.3%). Best overall response to 1L platinum-based chemotherapy was CR in six patients (20.0%), PR in 17 patients (56.7%), and SD in seven patients (23.3%). Response status at the start of avelumab treatment was CR in five patients (16.7%), PR in 18 patients (60.0%), and SD in seven patients (23.3%). The median treatment-free interval (TFI) between end of 1L chemotherapy and start of avelumab was 4.4 weeks (range, 1.0-21.0).

**Table 2:** Summary of prior treatment.

Characteristic	N=30
TFI prior to start of avelumab, weeks	
Mean (SD)	5.5 (4.2)
Median	4.4
Range	1.0-21.0
TFI prior to start of avelumab, n (%)	
<4 weeks	12 (40.0)
4 to <6 weeks	11 (36.7)
6 to <8 weeks	1 (3.3)
8 to 10 weeks	2 (6.7)
>10 weeks	4 (13.3)
1L platinum-based chemotherapy regimen, n (%)	
Cisplatin + gemcitabine	21 (70.0)
Carboplatin + gemcitabine	8 (26.7)
MVAC	1 (3.3)
No. of 1L chemotherapy cycles	
Mean (SD)	5.2 (1.0)
Median	5
Range	4-8
No. of 1L chemotherapy cycles, n (%)	
4	9 (30.0)
5	7 (23.3)
6	13 (43.3)
8	1 (3.3)
Best response to 1L chemotherapy, n (%)	
Complete response	6 (20.0)
Partial response	17 (56.7)
Stable disease	7 (23.3)
Response to 1L chemotherapy at the start of avelumab, n (%)	
Complete response	5 (16.7)
Partial response	18 (60.0)
Stable disease	7 (23.3)

1L, first line; MVAC, methotrexate, vinblastine, doxorubicin (Adriamycin), and cisplatin; SD, standard deviation; TFI, treatment-free interval.

Of 30 patients treated with avelumab, seven (23.3%) were still receiving avelumab despite the end of the EAP. The median duration of avelumab treatment was 6.2 months (range, 0.9-20.7), representing a median of 13.5 cycles (**Supplementary Table S3**). Nine patients received avelumab for >12 months; in this subgroup, the median treatment duration was 19.8 months (median, 42 cycles). Among patients who discontinued avelumab treatment (n=23 [76.7%]), reasons were PD (n=17 [73.9%]), AE (n=3 [13.0%]), and withdrawal of consent (n=3 [13.0%]; **Supplementary Table S1**). Fifteen patients received a subsequent anticancer therapy (50.0% of all patients; 65.0% of those who discontinued), most commonly rechallenge with platinum-based chemotherapy (**Supplementary Table S4**). The median time from the last avelumab administration to the start of subsequent anticancer therapy was 22 days (range, 15.0-97.0).

### 3.2 Safety

During avelumab treatment, TEAEs of any grade occurred in 27 patients (90.0%), including grade ≥3 TEAEs in eight (26.7%; **Table 3**; **Supplementary Table S5**). TRAEs were reported in 21 patients (70.0%), including grade ≥3 TRAEs in three patients (10.0%). The most common TRAEs of any grade were rash in four patients (13.3%) and pruritus in four patients (13.3%) (**Supplementary Table S6**). Infusion-related reactions occurred in three patients (10.0%) and none were grade ≥3. Serious TEAEs occurred in seven patients (23.3%) and serious TRAEs occurred in three patients (10.0%; comprising dyspnea, acute kidney injury, and deep vein thrombosis in one patient each; **Supplementary Table S7**). AEs led to interruption of avelumab treatment in 14 patients (46.7%) and discontinuation in three patients (10.0%). One patient died, which was due to PD and disease-related factors.

**Table 3:** Summary of adverse events during avelumab maintenance treatment.

Patients, n (%)	N=30	
	Any grade	Grade ≥3
TEAE	27 (90.0)	8 (26.7)
TRAE	21 (70.0)	3 (10.0)
Serious TEAE	7 (23.3)	7 (23.3)
Serious TRAE	3 (10.0)	3 (10.0)
Infusion-related reaction	3 (10.0)	0
TEAE leading to the interruption of avelumab	14 (46.7)	6 (20.0)
TEAE leading to the discontinuation of avelumab	3 (10.0)	2 (6.7)
TEAE resulting in death	1 (3.3)	1 (3.3)

TEAE, treatment-emergent adverse event; TRAE, treatment-related adverse event.

### 3.3 Effectiveness

**Figure 1:**
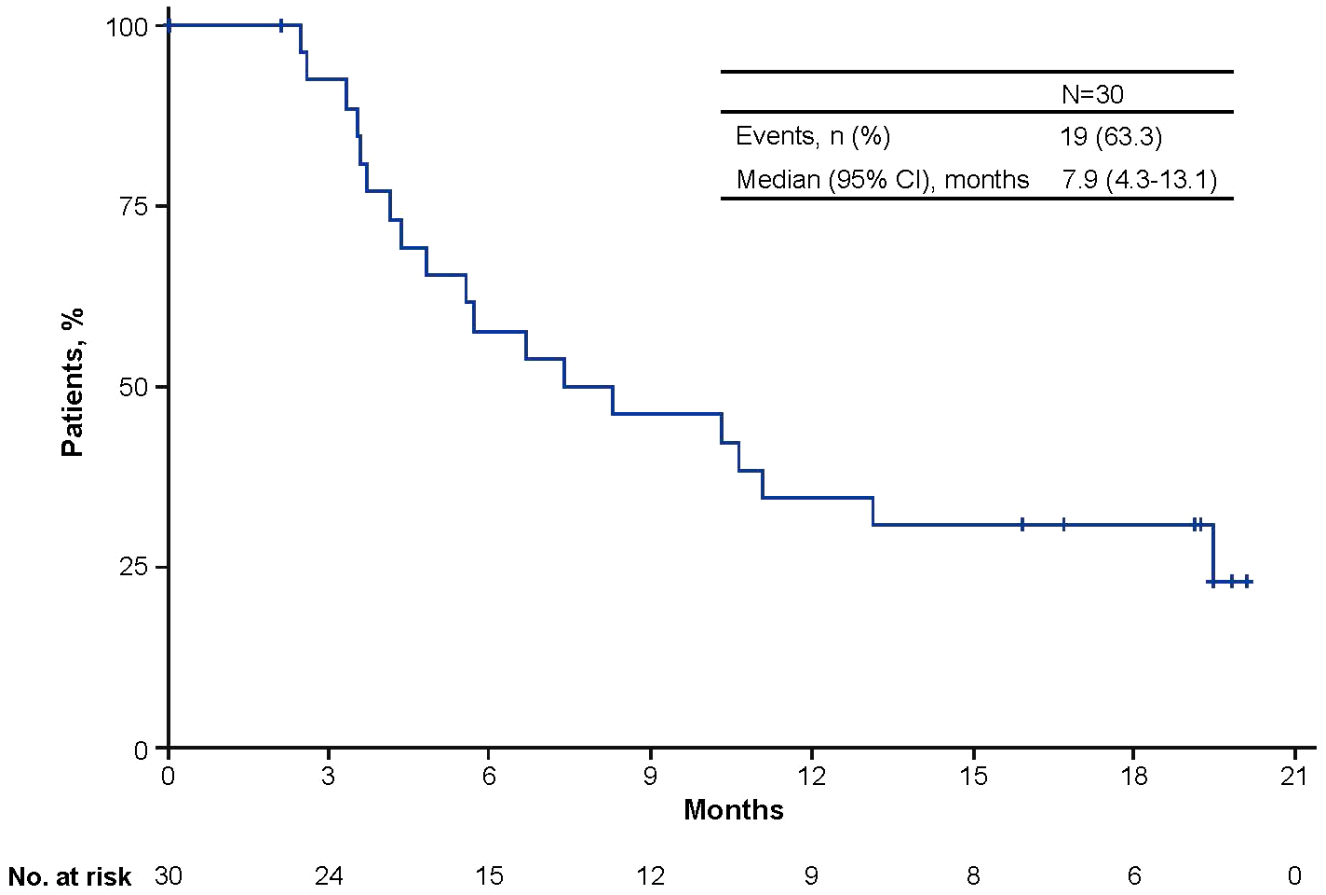
Kaplan-Meier analysis of progression-free survival from the start of avelumab maintenance treatment.

Median PFS from start of avelumab treatment was 7.9 months (95% CI, 4.3-13.1; **Figure 1**). OS was not analyzed because only one patient died. In the nine patients who received >12 months of avelumab treatment, median PFS was NE (95% CI, 1.3-NE; range, 13.1-20.1). During avelumab treatment, best overall response was CR in six patients (20.0%), PR in three patients (10.0%), SD in 14 patients (46.7%), PD in one patient (3.3%), and NE in six patients (20.0%; **Table 4**). The objective response rate was 30.0% (95% CI, 13.6-46.4). Among responding patients (CR or PR), median time to response was 2.7 months (range, 1.5-9.2) and median duration of response was 17.8 months (95% CI, 5.2-NE; range, 5.2-17.8).

**Table 4:** Summary of responses during avelumab maintenance treatment.

Category	N=30
Best overall response, n (%)	
Complete response	6 (20.0)
Partial response	3 (10.0)
Stable disease	14 (46.7)
Progressive disease	1 (3.3)
Not evaluable	6 (20.0)
Objective response rate, n (%)	9 (30.0)
(95% CI)	(13.6-46.4)
Disease control rate, n (%)	23 (76.7)
(95% CI)	(61.5-91.8)
Time to response, months	n=9
Mean (SD)	4.1 (3.1)
Median	2.7
Range	1.5-9.2
Duration of response	n=9
Events, n (%)	4 (44.4)
Median (95% CI), months*	17.8 (5.2-NE)
Range, months	5.2-17.8

NE, not evaluable; SD, standard deviation.

*Calculated using the Kaplan-Meier method.

## 4 Discussion

In the JAVELIN Bladder 100 phase 3 trial, avelumab 1L maintenance + BSC significantly prolonged both OS and PFS compared with BSC alone in patients with advanced UC who were progression free after 1L platinum-based chemotherapy, with long-term follow-up yielding consistent findings (18, 19). Comparable clinical outcomes and safety were reported in subsequent real-world studies performed in several countries; however, real-world data from Asian countries are limited (22–25). Here, we report the first real-world evidence for avelumab 1L maintenance treatment in Korean patients, including safety and effectiveness data, which was obtained from an EAP that enrolled patients prospectively.

In total, 30 patients were treated with avelumab. Although the number of patients was limited, baseline characteristics were mostly consistent with those seen in JAVELIN Bladder 100 and other real-world studies. For example, shared characteristics across all studies included a median age of >65 years, a higher proportion of men vs women, and a higher proportion of patients with bladder vs upper tract primary tumors (18, 22–26). Visceral metastases, which are an established negative prognostic factor in advanced UC (27), were present at diagnosis in 40% of patients in the EAP population. Nevertheless, in the JAVELIN Bladder 100 trial, HRs for OS and PFS favored avelumab 1L maintenance + BSC vs BSC alone in patients with visceral metastases (18, 19, 28). The most common 1L chemotherapy regimen in the EAP population was cisplatin + gemcitabine, consistent with the Asian subgroup of JAVELIN Bladder 100 (21). The number of cycles of 1L platinum-based chemotherapy received in the EAP population ranged from 4 to 6 in 97% of patients, which aligns with international guidelines and the design of the JAVELIN Bladder 100 study (3, 9, 10, 18). Because the optimal duration of prior 1L platinum-based chemotherapy is not yet defined, a phase 2 study assessing avelumab 1L maintenance treatment after 3 vs 6 cycles (DISCUS) is in progress (29).

Best response to 1L platinum-based chemotherapy in the EAP population was an objective response (CR or PR) in 77% and SD in 23%. In JAVELIN Bladder 100, OS and PFS benefits were demonstrated with avelumab 1L maintenance treatment in patients with CR, PR, or SD (19, 28). Patients enrolled in JAVELIN Bladder 100 were required to have a TFI of 4 to 10 weeks between end of chemotherapy and start of avelumab, which allowed any AEs to resolve and for tumor status to be evaluated (30). In the EAP population, TFIs were more heterogeneous (<4 weeks in 40%, 4 to 10 weeks in 47%, and >10 weeks in 13%). It should be noted that a TFI >10 weeks after completing 1L chemotherapy may increase the risk of disease progression (30). Overall, patient characteristics in this population are reflective of a real-world population of patients eligible for avelumab 1L maintenance treatment.

The median duration of avelumab treatment was 6.2 months. Seven patients (23%) continued treatment until the end of the EAP and nine patients (30%) received avelumab 1L maintenance treatment for >12 months. After initiating avelumab 1L maintenance treatment, grade ≥3 TRAEs and serious TRAEs occurred in 10% of patients, similar to previous studies (18, 19, 31). Overall, 70% of patients had a TRAE of any grade, most commonly low-grade skin disorders (23% of patients). No new safety signals were observed.

Median PFS in this population was 7.9 months, which is numerically higher than the PFS of 5.5 months reported after long-term follow-up in the JAVELIN Bladder 100 trial (19). In other real-world studies of avelumab 1L maintenance treatment, median PFS ranged from 5.7 to 9.6 months (22–24). In this EAP population, subgroup analyses of outcomes were not performed because of the small number of patients. In the JAVELIN Bladder 100 trial, OS and PFS benefits were seen with avelumab 1L maintenance treatment across a wide range of subgroups, including those defined by patient or disease characteristics, 1L chemotherapy regimen, response to 1L chemotherapy, duration of 1L chemotherapy, and TFI (18, 19, 28, 30). These data support the use of avelumab 1L maintenance treatment as the standard of care for platinum-treated patients without PD, irrespective of patient, disease, or prior treatment characteristics. An ongoing real-world study being conducted in the Asia-Pacific region (SPADE) will prospectively assess treatment outcomes with avelumab 1L maintenance, including 6- and 12-month OS and health-related quality of life (32).

Our study has several limitations, which should be considered when interpreting its findings. Firstly, the population size was relatively small (30 patients), and all patients received treatment at 5 institutions within a single country (Korea), which may limit the generalizability of the data reported. Study data only included information collected as part of routine clinical practice, which is less extensive than would have been collected in a clinical trial. Subgroup analyses could not be performed because of the small population size. Lastly, the duration of follow-up was too short to analyze overall survival.

In conclusion, our data provide the first real-world evidence of the clinical benefits and tolerable safety of avelumab 1L maintenance treatment in patients with advanced UC in Korea. Findings are comparable to those of the JAVELIN Bladder 100 trial and other real-world studies and further support the recommendation of avelumab 1L maintenance treatment as the standard of care for patients with advanced UC without PD after 1L platinum-based chemotherapy.

## Data availability statement

The datasets presented in this article are not readily available because of the need to maintain patient confidentiality. Any requests for data by qualified scientific and medical researchers for legitimate research purposes will be subject to Merck’s Data Sharing Policy. All requests should be submitted in writing to Merck’s data sharing portal (https://www.merckgroup.com/en/research/our-approach-to-research-and-development/healthcare/clinical-trials/commitment-responsible-data-sharing.html). When Merck has a co-research, co-development, or co-marketing or co-promotion agreement, or when the product has been out-licensed, the responsibility for disclosure might be dependent on the agreement between parties. Under these circumstances, Merck will endeavor to gain agreement to share data in response to requests.

## Ethics statement

This expanded access program was performed in accordance with ethical principles that have their origin in the Declaration of Helsinki and are consistent with International Council for Harmonisation and Good Clinical Practice and Ministry of Food and Drug Safety requirements. The protocol, amendments, and informed consent form were approved by institutional review boards at each site. Written informed consent was obtained from all patients. 

## Author contributions

SHP: Investigation, Resources, Writing – review & editing. SJS: Investigation, Resources, Writing – review & editing. SYR: Investigation, Resources, Writing – review & editing. S-HB: Investigation, Resources, Writing – review & editing. HKS: Investigation, Resources, Writing – review & editing. BK: Investigation, Resources, Writing – review & editing. MK: Investigation, Resources, Writing – review & editing. Y-HH: Data curation, Formal Analysis, Project administration, Writing – original draft, Writing – review & editing. SY: Investigation, Resources, Writing – review & editing. J-LL: Investigation, Resources, Writing – review & editing.
