# Supplementary material for: Avelumab first-line maintenance treatment in patients with locally advanced or metastatic urothelial carcinoma: real-world results from a Korean expanded access program
Source: Front Oncol. 2024 Jun 3;14:1403120. doi: 10.3389/fonc.2024.1403120 (PMC11180756; doi:10.3389/fonc.2024.1403120)
Supplement: Supplementary file 1 [file Table_1.docx]

**SUPPLEMENTARY APPENDIX**

# Table S1. Patient disposition

| Provided informed consent, n | 31 |
| --- | --- |
| Received avelumab treatment, n (%) | 30 (96.8) |
| Did not receive avelumab treatment, n (%)* | 1 (3.2) |
| Treatment status at the end of the program, n (%) | n=30 |
| Still receiving avelumab | 7 (23.3) |
| Discontinued avelumab | 23 (76.7) |
| Primary reason for discontinuing avelumab, n (%) | n=23 |
| Progression of underlying disease | 17 (73.9) |
| Adverse event | 3 (13.0) |
| Withdrawal of informed consent | 3 (13.0) |
| Completed the program, n (%) | n=30 |
| Yes | 25 (83.3) |
| No | 5 (16.7) |
| Primary reason for discontinuing from the program, n (%) | n=5 |
| Progressive disease | 1 (20.0) |
| Withdrawal of informed consent | 3 (60.0) |
| Death | 1 (20.0) |

*This patient did not receive avelumab because they did not meet the eligibility criterion requiring lack of disease progression after first-line platinum-based chemotherapy.

# Table S2. Surgical history in the study population.

| **Patients, n (%)** | **N=30** |
| --- | --- |
| Transurethral bladder resection | 18 (60.0) |
| Radical cystectomy | 7 (23.3) |
| Nephroureterectomy | 4 (13.3) |
| Nephrectomy | 2 (6.7) |
| Transurethral prostatectomy | 2 (6.7) |
| Lymphadenectomy | 1 (3.3) |
| Renal exploration | 1 (3.3) |
| Tumor excision | 1 (3.3) |
| Ureterectomy | 1 (3.3) |
| Ureteric operation | 1 (3.3) |
| Urethrectomy | 1 (3.3) |
| Urinary cystectomy | 1 (3.3) |

# Table S3. Exposure to avelumab.

| **Parameter** | **N=30** |
| --- | --- |
| Duration of treatment, months |  |
| Mean (SD) | 9.2 (7.1) |
| Median | 6.2 |
| Range | 0.9-20.7 |
| Number of cycles |  |
| Mean (SD) | 19.4 (15.2) |
| Median | 13.5 |
| Range | 2-43 |
| Cumulative dose, mg/kg* |  |
| Mean (SD) | 193.8 (151.2) |
| Median | 135.0 |
| Range | 20.0-440.4 |
| Dose intensity, mg/kg/cycle^†^ |  |
| Mean (SD) | 10.0 (0.2) |
| Median | 10.0 |
| Range | 9.6-10.5 |

SD, standard deviation.
*Sum of single dose (mg) per kg of body weight before dosing for each cycle received.
^†^Cumulative dose (mg/kg) divided by total number of cycles.

# Table S4. Subsequent anticancer therapy after discontinuation of avelumab

| **Parameter** | **N=30** |
| --- | --- |
| Received subsequent anticancer therapy, n (%) |  |
| Yes | 15 (50.0) |
| No | 15 (50.0) |
| Type of subsequent anticancer therapy in any line, n (%)* | n=15 |
| Rechallenge with platinum-based chemotherapy | 8 (53.3) |
| PD-1 or PD-L1 inhibitor | 3 (20.0) |
| Other anticancer therapy | 9 (60.0) |
| Type of anticancer therapy received as second-line treatment, n (%) | n=15 |
| Rechallenge with platinum-based chemotherapy | 6 (40.0) |
| PD-1 or PD-L1 inhibitor | 1 (6.7) |
| Other anticancer therapy | 8 (53.3) |
| Time to initiation of second-line anticancer therapy, days | n=15 |
| Mean (SD) | 31.7 (22.2) |
| Median | 22.0 |
| Range | 15-97 |

PD-1, programmed death protein 1; PD-L1, programmed death ligand 1; SD, standard deviation.
*Patients who received >1 type of anticancer therapy in different lines (n=6) are counted in all relevant categories; one patient who received combination treatment (pembrolizumab + pemetrexed) is counted in both relevant categories (“PD-1 or PD-L1 inhibitor” and “other anticancer therapy”).

# Table S5. Summary of treatment-emergent adverse events (N=30)

|  | **Any grade** |  | **Grade ≥3** |  |
| --- | --- | --- | --- | --- |
| **System organ class**  **Preferred term** | **Number of patients, n (%)** | **Number of occurrences, n** | **Number of patients, n (%)** | **Number of occurrences, n** |
| Any event | 27 (90.0) | 83 | 8 (26.7) | 10 |
| Gastrointestinal disorders | 8 (26.7) | 9 | 1 (3.3) | 1 |
| Abdominal pain | 2 (6.7) | 2 | 0 | 0 |
| Constipation | 2 (6.7) | 2 | 0 | 0 |
| Diarrhea | 2 (6.7) | 2 | 0 | 0 |
| Dry mouth | 1 (3.3) | 1 | 0 | 0 |
| Dysphagia | 1 (3.3) | 1 | 0 | 0 |
| Ileus | 1 (3.3) | 1 | 1 (3.3) | 1 |
| General disorders and administration site conditions | 7 (23.3) | 9 | 0 | 0 |
| Fatigue | 3 (10.0) | 4 | 0 | 0 |
| Feeling cold | 1 (3.3) | 1 | 0 | 0 |
| Edema | 1 (3.3) | 1 | 0 | 0 |
| Edema peripheral | 1 (3.3) | 1 | 0 | 0 |
| Pain | 1 (3.3) | 1 | 0 | 0 |
| Pyrexia | 1 (3.3) | 1 | 0 | 0 |
| Skin and subcutaneous tissue disorders | 7 (23.3) | 9 | 0 | 0 |
| Rash | 4 (13.3) | 5 | 0 | 0 |
| Pruritus | 4 (13.3) | 4 | 0 | 0 |
| Infections and infestations | 7 (23.3) | 8 | 3 (10.0) | 3 |
| COVID-19 | 4 (13.3) | 4 | 0 | 0 |
| Herpes zoster | 1 (3.3) | 1 | 0 | 0 |
| Pneumonia | 1 (3.3) | 1 | 1 (3.3) | 1 |
| Pyelonephritis acute | 1 (3.3) | 1 | 1 (3.3) | 1 |
| Urinary tract infection | 1 (3.3) | 1 | 1 (3.3) | 1 |
| Nervous system disorders | 6 (20.0) | 7 | 0 | 0 |
| Peripheral motor neuropathy | 2 (6.7) | 2 | 0 | 0 |
| Dizziness | 1 (3.3) | 1 | 0 | 0 |
| Headache | 1 (3.3) | 1 | 0 | 0 |
| Neuropathy peripheral | 1 (3.3) | 1 | 0 | 0 |
| Paresthesia | 1 (3.3) | 1 | 0 | 0 |
| Peripheral sensory neuropathy | 1 (3.3) | 1 | 0 | 0 |
| Renal and urinary disorders | 6 (20.0) | 6 | 2 (6.7) | 2 |
| Acute kidney injury | 2 (6.7) | 2 | 2 (6.7) | 2 |
| Pollakiuria | 2 (6.7) | 2 | 0 | 0 |
| Micturition urgency | 1 (3.3) | 1 | 0 | 0 |
| Nocturia | 1 (3.3) | 1 | 0 | 0 |
| Investigations | 5 (16.7) | 6 | 0 | 0 |
| SARS-CoV-2 test positive | 3 (10.0) | 3 | 0 | 0 |
| Alanine aminotransferase increased | 1 (3.3) | 1 | 0 | 0 |
| Aspartate aminotransferase increased | 1 (3.3) | 1 | 0 | 0 |
| Platelet count decreased | 1 (3.3) | 1 | 0 | 0 |
| Respiratory, thoracic, and mediastinal disorders | 5 (16.7) | 5 | 2 (6.7) | 2 |
| Cough | 3 (10.0) | 3 | 0 | 0 |
| Dyspnea | 1 (3.3) | 1 | 1 (3.3) | 1 |
| Emphysema | 1 (3.3) | 1 | 1 (3.3) | 1 |
| Musculoskeletal and connective tissue disorders | 4 (13.3) | 5 | 0 | 0 |
| Flank pain | 3 (10.0) | 3 | 0 | 0 |
| Arthralgia | 1 (3.3) | 1 | 0 | 0 |
| Pain in extremity | 1 (3.3) | 1 | 0 | 0 |
| Metabolism and nutrition disorders | 4 (13.3) | 4 | 1 (3.3) | 1 |
| Decreased appetite | 3 (10.0) | 3 | 0 | 0 |
| Hyperkalemia | 1 (3.3) | 1 | 1 (3.3) | 1 |
| Endocrine disorders | 3 (10.0) | 5 | 0 | 0 |
| Hyperthyroidism | 3 (10.0) | 3 | 0 | 0 |
| Hypothyroidism | 2 (6.7) | 2 | 0 | 0 |
| Injury, poisoning, and procedural complications | 3 (10.0) | 3 | 0 | 0 |
| Infusion-related reaction | 3 (10.0) | 3 | 0 | 0 |
| Vascular disorders | 1 (3.3) | 2 | 1 (3.3) | 1 |
| Deep vein thrombosis | 1 (3.3) | 1 | 1 (3.3) | 1 |
| Lymphoedema | 1 (3.3) | 1 | 0 | 0 |
| Blood and lymphatic system disorders | 1 (3.3) | 1 | 0 | 0 |
| Eosinophilia | 1 (3.3) | 1 | 0 | 0 |
| Ear and labyrinth disorders | 1 (3.3) | 1 | 0 | 0 |
| Tinnitus | 1 (3.3) | 1 | 0 | 0 |
| Eye disorders | 1 (3.3) | 1 | 0 | 0 |
| Vision blurred | 1 (3.3) | 1 | 0 | 0 |
| Hepatobiliary disorders | 1 (3.3) | 1 | 0 | 0 |
| Cholangitis | 1 (3.3) | 1 | 0 | 0 |
| Psychiatric disorders | 1 (3.3) | 1 | 0 | 0 |
| Insomnia | 1 (3.3) | 1 | 0 | 0 |

# Table S6. Summary of treatment-related adverse events (N=30)

|  | **Any grade** |  | **Grade ≥3** |  |
| --- | --- | --- | --- | --- |
| **System organ class**  **Preferred term** | **Number of patients, n (%)** | **Number of occurrences, n** | **Number of patients, n (%)** | **Number of occurrences, n** |
| Any event | 21 (70.0) | 42 | 3 (10.0) | 3 |
| Skin and subcutaneous tissue disorders | 7 (23.3) | 9 | 0 | 0 |
| Rash | 4 (13.3) | 5 | 0 | 0 |
| Pruritus | 4 (13.3) | 4 | 0 | 0 |
| General disorders and administration site conditions | 6 (20.0) | 8 | 0 | 0 |
| Fatigue | 3 (10.0) | 4 | 0 | 0 |
| Feeling cold | 1 (3.3) | 1 | 0 | 0 |
| Edema | 1 (3.3) | 1 | 0 | 0 |
| Pain | 1 (3.3) | 1 | 0 | 0 |
| Pyrexia | 1 (3.3) | 1 | 0 | 0 |
| Gastrointestinal disorders | 4 (13.3) | 4 | 0 | 0 |
| Constipation | 2 (6.7) | 2 | 0 | 0 |
| Diarrhea | 1 (3.3) | 1 | 0 | 0 |
| Dry mouth | 1 (3.3) | 1 | 0 | 0 |
| Endocrine disorders | 3 (10.0) | 5 | 0 | 0 |
| Hyperthyroidism | 3 (10.0) | 3 | 0 | 0 |
| Hypothyroidism | 2 (6.7) | 2 | 0 | 0 |
| Injury, poisoning, and procedural complications | 3 (10.0) | 3 | 0 | 0 |
| Infusion-related reaction | 3 (10.0) | 3 | 0 | 0 |
| Investigations | 3 (10.0) | 3 | 0 | 0 |
| Alanine aminotransferase increased | 1 (3.3) | 1 | 0 | 0 |
| Platelet count decreased | 1 (3.3) | 1 | 0 | 0 |
| SARS-CoV-2 test positive | 1 (3.3) | 1 | 0 | 0 |
| Nervous system disorders | 3 (10.0) | 3 | 0 | 0 |
| Dizziness | 1 (3.3) | 1 | 0 | 0 |
| Neuropathy peripheral | 1 (3.3) | 1 | 0 | 0 |
| Peripheral motor neuropathy | 1 (3.3) | 1 | 0 | 0 |
| Respiratory, thoracic, and mediastinal disorders | 3 (10.0) | 3 | 1 (3.3) | 1 |
| Cough | 2 (6.7) | 2 | 0 | 0 |
| Dyspnea | 1 (3.3) | 1 | 1 (3.3) | 1 |
| Ear and labyrinth disorders | 1 (3.3) | 1 | 0 | 0 |
| Tinnitus | 1 (3.3) | 1 | 0 | 0 |
| Musculoskeletal and connective tissue disorders | 1 (3.3) | 1 | 0 | 0 |
| Arthralgia | 1 (3.3) | 1 | 0 | 0 |
| Renal and urinary disorders | 1 (3.3) | 1 | 1 (3.3) | 1 |
| Acute kidney injury | 1 (3.3) | 1 | 1 (3.3) | 1 |
| Vascular disorder | 1 (3.3) | 1 | 1 (3.3) | 1 |
| Deep vein thrombosis | 1 (3.3) | 1 | 1 (3.3) | 1 |

# Table S7. Summary of serious TEAEs and serious TRAEs (N=30).

|  | **Serious TEAE** |  | **Serious TRAE** |  |
| --- | --- | --- | --- | --- |
| **System organ class**  **Preferred Term** | **Number of patients, n (%)** | **Number of occurrences, n** | **Number of patients, n (%)** | **Number of occurrences, n** |
| Total | 7 (23.3) | 8 | 3 (10.0) | 3 |
| Infections and infestations | 3 (10.0) | 3 | 0 | 0 |
| Pneumonia | 1 (3.3) | 1 | 0 | 0 |
| Pyelonephritis acute | 1 (3.3) | 1 | 0 | 0 |
| Urinary tract infection | 1 (3.3) | 1 | 0 | 0 |
| Respiratory, thoracic, and mediastinal disorders | 2 (6.7) | 2 | 1 (3.3) | 1 |
| Dyspnea | 1 (3.3) | 1 | 1 (3.3) | 1 |
| Emphysema | 1 (3.3) | 1 | 0 | 0 |
| Gastrointestinal disorders | 1 (3.3) | 1 | 0 | 0 |
| Ileus | 1 (3.3) | 1 | 0 | 0 |
| Renal and urinary disorders | 1 (3.3) | 1 | 1 (3.3) | 1 |
| Acute kidney injury | 1 (3.3) | 1 | 1 (3.3) | 1 |
| Vascular disorders | 1 (3.3) | 1 | 1 (3.3) | 1 |
| Deep vein thrombosis | 1 (3.3) | 1 | 1 (3.3) | 1 |

TEAE, treatment-emergent adverse event; TRAE, treatment-related adverse event.
